# The effect of the time interval between coronary angiography and on-pump cardiac surgery on risk of postoperative acute kidney injury: a meta-analysis

**DOI:** 10.1186/1749-8090-8-178

**Published:** 2013-08-03

**Authors:** Yijie Hu, Zhiping Li, Jianming Chen, Cheng Shen, Yi Song, Qianjin Zhong

**Affiliations:** 1 Department of Cardiovascular Surgery, Institute of Surgery Research, Daping Hospital, Third Military Medical University, No. 10 Changjiang Zhi Road, Yuzhong District, Chongqing 400042, China

**Keywords:** Acute kidney injury, Coronary angiography, Cardiac surgery

## Abstract

**Background:**

Reports of the association between the time interval from coronary angiography (CAG) to cardiac surgery and risk of postoperative acute kidney injury (AKI) are controversial. We attempted to examine this association by conducting a meta-analysis.

**Methods:**

We searched the Pubmed, MEDLINE, EMBASE, Web of Science databases, and the Cochrane Library from January 1966 to March 2013. A meta-analysis of studies reporting data for 1-day and 3-day time intervals between CAG and cardiac surgery was conducted after evaluation of heterogeneity and publication bias. Study-specific estimates were combined with inverse variance-weighted averages of logarithmic odds ratios (ORs) in fixed-effects models.

**Results:**

From 8 studies involving 11542 persons, the pooled OR of AKI associated with an interval of 1 day or less between CAG and surgery was 1.21 (95% confidence interval (CI), 1.04 to 1.39) relative to an interval of more than 1 day. From 4 studies involving 5420 persons in the cardiopulmonary-bypass subgroup, the pooled OR of AKI associated with an interval of 3 days or less between CAG and surgery was 1.25 (95% CI, 1.07 to 1.43) relative to an interval of more than 3 days. The adjusted OR of the study in the cardiopulmonary bypass/ deep hypothermic circulatory arrest subgroup was 0.35 (95% CI, 0.17 to 0.73).

**Conclusions:**

A time interval of 1 day or less between CAG and on-pump cardiac surgery was significantly associated with increased risk of AKI. A delay of on-pump cardiac surgery until 24 hours after CAG can potentially decrease postoperative AKI.

## Background

Postoperative acute kidney injury (AKI) is one of the most serious and frequent complications of cardiac surgery. Previous studies have demonstrated that even small increases in serum creatinine following cardiac surgery are independently associated with increased mortality and longer hospitalization [1-3]. As no causal therapy for AKI is currently available, every effort has to be made to prevent AKI [4].

Lately it has become common practice to provide same admission [5] or even one-stage diagnostic coronary angiography (CAG) and surgical services for patients undergoing cardiovascular surgery. The types of surgeries mainly include coronary artery bypass grafting (CABG), aortic surgery, and valve surgery. AKI is reported to occur in up to 30% of patients after on-pump cardiac surgery [6,7], while contrast-induced nephropathy after CAG occurs in up to 10% of patients with normal renal function and up to 25% of patients with pre-existing renal impairment [8]. Hence, many studies have dealt with the question of whether the closely spaced “double hit” on renal function increases the risk of postoperative AKI. Conflicting data have been reported. Some authors claimed that the risk of AKI after cardiac surgery is not influenced by the time interval between angiography and cardiac surgery [9-13]. Conversely, other authors emphasized the deleterious effect of performing both procedures in close succession [14-17].

In view of the limited clarity of the available data, we conducted a systematic review of the literature and a meta-analysis of selected studies to evaluate the effect of the time interval between CAG and cardiac surgery on risk of postoperative AKI.

## Methods

### Search strategy

The keywords used to search included the following: “cardiac surgery or CABG or valve surgery or aortic surgery” and “catheterization or angiography or percutaneous coronary intervention” and "acute kidney injury or AKI or renal failure." A computerized search of the Pubmed, MEDLINE, EMBASE, Web of Science databases, and the Cochrane Library from January 1966 to March 2013 was undertaken to identify potentially eligible studies on the basis of the title, abstract, and keywords; no language limitation was applied. Then, the full content of each article was examined to decide which studies met the inclusion and exclusion criteria mentioned in the next section. The reference lists from all studies, narrative reviews, and systematic reviews identified by electronic searches were manually searched to identify additional eligible studies. Two authors (Yijie Hu and Zhiping Li) independently performed the eligibility assessments; if opinions differed, the differences were resolved by consensus.

### Inclusion and exclusion criteria

Included studies met the following criteria: (1) the study focused on the risk of AKI and the time interval between angiography and cardiac surgery; (2) the study was a randomized controlled trial, case-control, or cohort study; (3) the study either provided risk estimates with the odds ratio (OR), and 95% confidence interval (95% CI), or sufficient information was available to calculate the OR and 95% CI.

A study was excluded from the meta-analysis if (1) it only provided an effect estimate but no means to calculate a 95% CI; (2) it did not provide an accurate definition of AKI; (3) it did not provide an accurate time interval; (4) off-pump heart surgery was performed, but the combined AKI risk due to contrast media and cardiopulmonary bypass (CPB) could not be evaluated; or (5) it was a low-quality study. In the case of multiple studies with the same or overlapping data published by the same researchers, we selected the most recent study with the largest number of participants.

### Data extraction

For each study, two authors (Yijie Hu and Yi Song) extracted the following data: the first author’s surname, country the study was conducted in, year reported, study design, sample size, primary operation, definition of AKI, effect estimate (95% CI), and adjusted covariates. If the effect estimate could be acquired from the searched results of the tabulated literature, they were extracted carefully from all eligible publications, which met the inclusion criteria. If data were not directly available, they were calculated from the published positive predictive values and/or the negative predictive values when appropriate. If a study contained unclear or incomplete information, the reviewers contacted the original authors for verification. Differences in data extraction were resolved by a third reviewer, referring back to the original article.

### Quality evaluation

We applied the Newcastle-Ottawa scale (NOS) [18] to evaluate the qualities of the included studies. A “star system” was used to judge the data quality of these studies on the basis of three broad categories: the selection, the comparability, and the outcome or exposure of interest. The stars were summed to compare the quality of a study in a quantitative fashion. The scores ranged from 0 to 9 stars. Studies with scores of 6 stars or greater were considered to be of high quality studies. Two reviewers (Yijie Hu and Yi Song) independently evaluated and cross-checked the qualities of the included studies, and assessed the bias of the studies. An open discussion was held to confirm the scores of those studies that received a different score from each reviewer.

### Statistical analysis

For each study, data regarding the incidence of AKI were used to generate ORs and 95% CI; or the adjusted ORs and 95% CI were extracted directly. According to time intervals reported in the literature, two meta-analyses were conducted: one analysis for interval of 1 day or less (the <1-day group), and one analysis for interval of 3 days or less ( the <3-day group). Among the studies of each group, AKI was mainly induced by contrast and ischemia-reperfusion injury after cardiopulmonary bypass, with the exception of the only study that used deep hypothermic circulatory arrest (DHCA), which we reported separately. All studies included in the subgroup analysis are functionally identical, and the effect size in our meta-analysis differ mainly because of sampling error. Accordingly the pooled OR estimates were combined by using inverse variance-weighted averages of logarithmic ORs in a fixed-effects model (the Mantel–Haenszel method). Heterogeneity among studies was determined by the chi-square-based Q test and the I^2^ statistics. A *P* value of less than .05 for the Q test and an I^2^ value of greater than 50% were considered as a measure of severe heterogeneity. A funnel plot was constructed to determine if publication bias existed and to examine differences between the effects in large and small studies; the studies were also assessed by applying Egger’s weighted regression test. The Egger’s test was also applied to assess less than 6 studies, with a P value of < 0.05 indicating significant publication bias among the included studies. The effects sizes, given as the OR on a logarithmic scale, were plotted against a measure of precision expressed as the inverse standard error. All statistical analyses were performed by using Stata Statistical Software (Version 11.0; StataCorp LP, Texas, USA).

## Results

### Description of the studies

**Figure 1:**
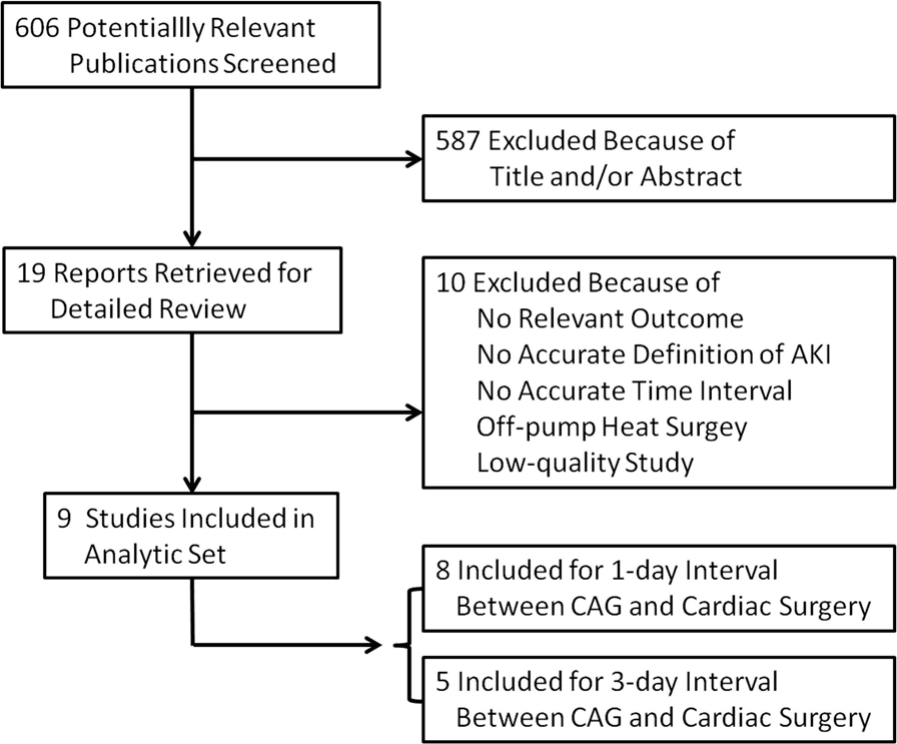
**Flow diagram of study selection for the meta-analysis.** AKI = acute kidney injury; CAG = coronary angiography.

As outlined in Figure 1, we identified 9 studies for the meta-analysis, including 5 cohort studies and 4 case-control studies. Detailed characteristics of the studies are listed in Table 1. Among the 9 studies, 5 studies used the AKI Network definition of AKI [19], an absolute increase in serum creatinine to ≥0.3 mg/dL, or a relative increase of ≥50% from the baseline value within 48 h after surgery, or a requirement for postoperative dialysis; 3 studies defined AKI on the basis of the RIFLE (Risk, Injury, Failure, Loss, End-stage renal disease) criteria [20] (“R” stage: plasma creatinine levels ≥1.5 × baseline; “I” stage: plasma creatinine levels ≥2.0 × baseline); and 1 study defined AKI as a greater than 25% rise in serum creatinine by the third postoperative day or as renal dysfunction that required the initiation of dialysis. Quality assessment of all studies was performed by using the NOS method (Table 2). The assessments ranged from a star rating of 6 to 8 (mean star rating, 7) with a higher value indicating better methodology.

**Table 1:** Main characteristics of 9 included studies

**Study**	**Year**	**Country**	**Type of study**	**Sample size**	**Main operation**	**Definition of AKI**	**Total AKI incidence**	**Adjustment**
Ranucci [14]	2013	USA	Cohort	4440	CABG	A	21.7%	Age, EF, MI, congestive heart failure, previous operation, urgent operation, creatinine, CPB duration, nadir HCT
					Valve Surgery			
Ko [9]	2012	USA	Cohort	2133	CABG	A	32.0%	None
					AVR			
Mcllroy [21]	2012	USA	Cohort	644	CABG	A	21.9%	Age, BMI, CPB duration, procedure type, N-acetylcysteine administration, modified EuroSCORE
					Valve Surgery			
Greason [11]	2012	USA	Cohort	642	AVR	A	22.7%	None
Andersen [10]	2012	USA	Case-control	285	Aorta replacement	B (R)	31.0%	Age, sex, BMI, eGFR, hypertension, CHF, diabetes, CPB duration, EF, hemoglobin, aprotinin exposure
					CABG			
Mehta [22]	2011	USA	Case-control	2441	CABG	A	17.1%	Age, sex, race, BMI, diabetes, CHF, MI, EF, hypertension, contrast volume and type, cardiogenic shock, cross-clamp time, creatinine, hemoglobin
Medalion [17]	2010	Israel	Case-control	395	CABG	B (R)	13.6%	None
Hennessy [16]	2010	USA	Cohort	197	Valve Surgery	B (I)	6.6%	None
					CABG			
Del Duca [15]	2007	Canada	Case-control	649	CABG	C	24.0%	Age, CPB duration, baseline GFR
					Valve Surgery			

A: defined by the AKI network: absolute increase of ≥ 0.3 mg/dL or a relative increase of ≥ 50% in serum creatinine from baseline value within 48 h after surgery, or a requirement for postoperative dialysis.

B: defined by the Risk, Injury, Failure, Loss, End-stage renal disease (RIFLE) criteria for acute renal failure, which are based on differences between the baseline and peak postoperative serum creatinine levels (“R” stage: plasma creatinine level ≥ 1.5 × baseline; “I” stage: plasma creatinine level ≥ 2.0 × baseline).

C: defined as a rise in serum creatinine of greater than 25% by the third postoperative day or renal dysfunction that requires the initiation of dialysis.

Abbreviations, *AKI* acute kidney injury, *AVR* aortic valve replacement, *BMI* body mass index; *CABG* coronary artery bypass grafting, *CHF* congestive heart failure, *CPB* cardiopulmonary bypass, *DHCA* deep hypothermic circulatory arrest, *EF* ejection fraction, *eGFR* estimated glomerular filtration rate, *GFR* glomerular filtration rate, *HCT* hematocrit, *IMA* internal mammary artery, *MI* myocardial infarction.

**Table 2:** Assessment of study quality

**Study**	**Quality indicators from the Newcastle-Ottawa scale**											**Score**
	**Selection**					**Comparability**			**Exposure/Outcome**			
	1	2	3	4		5a	5b		6	7	8	
Ranucci [14]	Yes	Yes	No	Yes		No	No		Yes	Yes	Yes	6
Ko [9]	Yes	Yes	No	Yes		No	No		Yes	Yes	Yes	6
Mcllroy [21]	Yes	Yes	No	Yes		Yes	Yes		Yes	Yes	Yes	8
Greason [11]	Yes	No	No	Yes		Yes	Yes		Yes	Yes	Yes	7
Andersen [10]	Yes	Yes	No	Yes		No	Yes		Yes	Yes	Yes	7
Mehta [22]	Yes	Yes	No	Yes		Yes	Yes		Yes	Yes	Yes	8
Medalion [17]	Yes	Yes	No	Yes		Yes	No		Yes	Yes	Yes	7
Hennessy [16]	Yes	No	No	Yes		Yes	Yes		Yes	Yes	Yes	7
Del Duca [15]	No	Yes	No	No		Yes	Yes		Yes	Yes	Yes	6

There were 8 studies of a 1-day time interval between CAG and cardiac surgery, and 5 studies of a 3-day time interval.

### Meta-analysis of studies reporting data for a 1-day time interval between CAG and cardiac surgery

**Figure 2:**
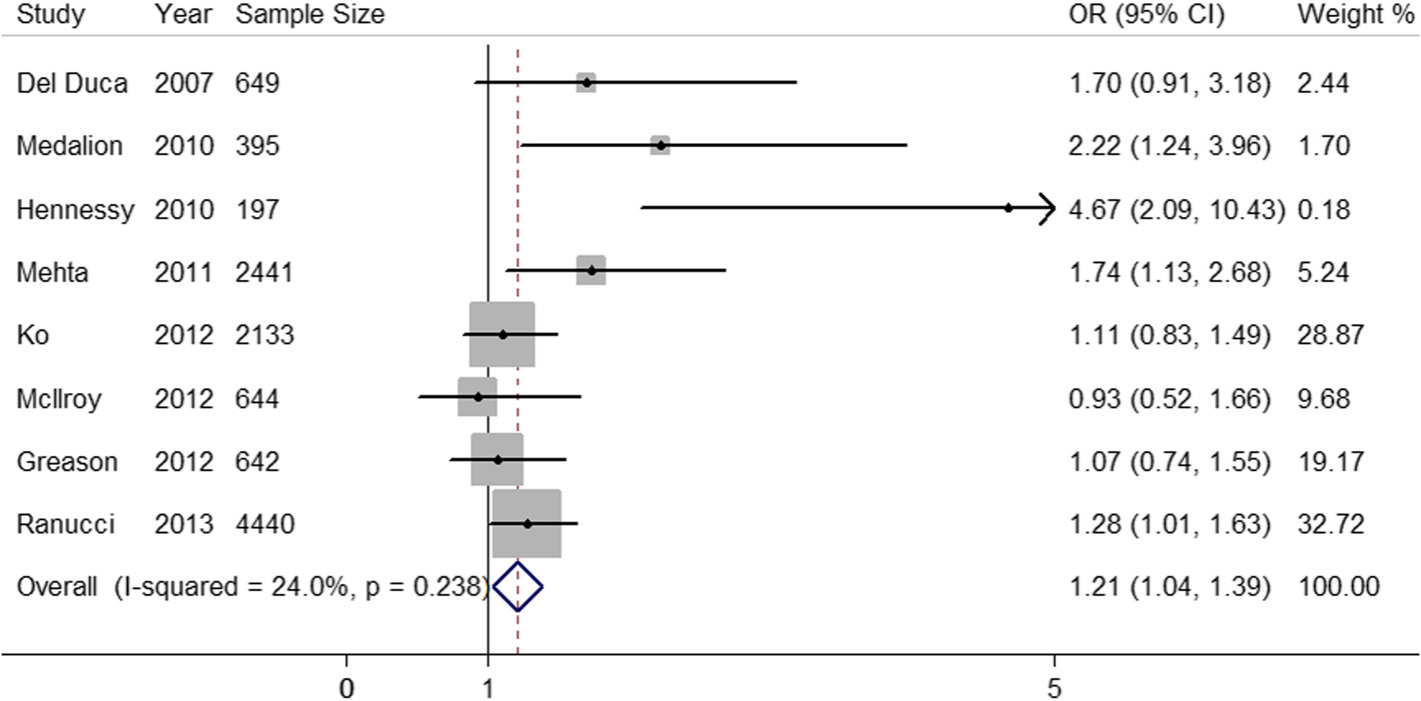
**Forest plot for time interval, ≤1-day vs >1 day.** The estimated odds ratio (OR) of each individual article corresponds to the middle of the squares, and the horizontal line gives the 95% confidence interval (CI). The sum of the statistics along with the summary OR is represented by the middle of the solid diamonds. The heterogeneity test statistic (I statistic) between articles is given below the summary statistics.

**Figure 3:**
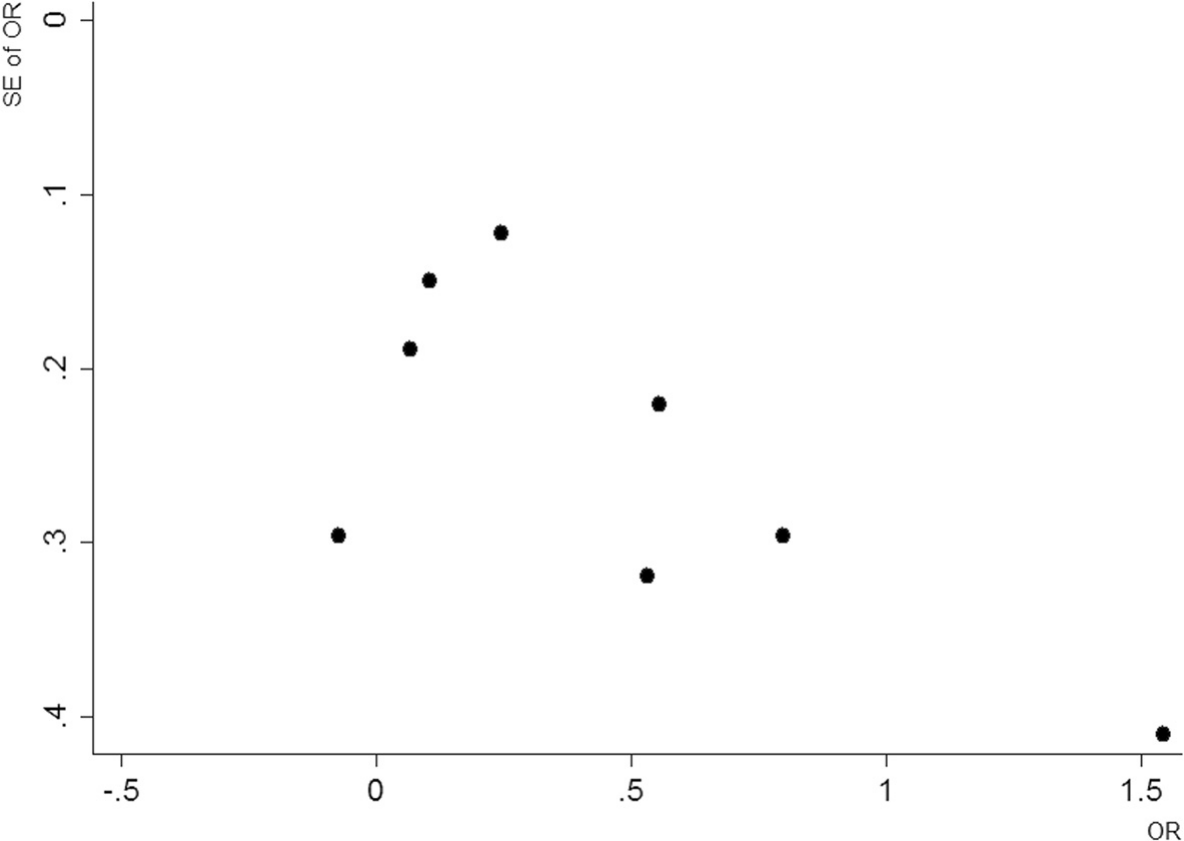
Funnel plot for studies of a 1-day time interval.

Four of the 8 individual studies demonstrated a statistically significant effect of a ≤ 1-day time interval on the incidence of AKI. Pooled analysis of the 8 studies revealed a significant increase in AKI risk by a factor of 1.21 with a ≤1-day time interval relative to >1 day in fixed-effects models (Figure 2). There was minimal trial heterogeneity (I^2^ = 24.0%, *P* = 0.238). Assessment of publication bias by visual examination of the funnel plot (Figure 3) and by application of Egger’s weighted regression test (*P* = 0.102) indicated no significant publication bias.

### Meta-analysis of studies reporting data for a 3-day time interval between CAG and cardiac surgery

The 5 studies of a 3-day time interval exhibited severe heterogeneity (I^2^ = 86.7%, *P* < 0.01). After comparing the basic and clinical characteristics of the 5 studies, we divided them into two subgroups: one (the CPB subgroup) included 4 studies that did not use deep hypothermic circulatory arrest (DHCA), which is a significant risk factor of AKI [23,24]; and the other (the CPB/DHCA subgroup) included only one study that did use DHCA. Meta-regression was not further performed due to the limited number of available studies.

**Figure 4:**
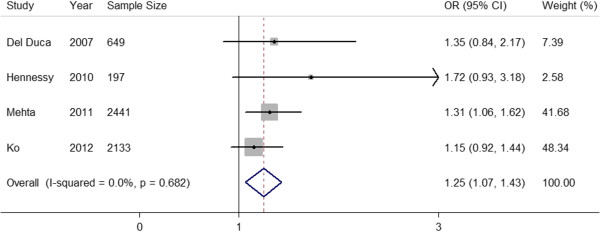
**Forest plot for time interval, ≤3-day vs >3 days, in CPB subgroup.** The estimated odds ratio (OR) of each individual article corresponds to the middle of the squares, and the horizontal line gives the 95% confidence interval (CI).The sum of the statistics along with the summary OR is represented by the middle of the solid diamonds. The heterogeneity test statistic (I statistic) between articles is given below the summary statistics.

In the CPB subgroup, only 1 of the 4 studies demonstrated a statistically significant effect of a ≤ 3-day time interval between CAG and cardiac surgery on the incidence of AKI. Pooled analysis of the 4 studies revealed a significant increase in AKI risk, by a factor of 1.25, with a ≤ 3-day time interval relative to > 3 days in fixed-effects models (Figure 4). The 4 studies of this subgroup exhibited no heterogeneity (I^2^ = 0%, *P* = 0.682). In addition, Egger’s test revealed no evidence of significant publication bias (*P* = 0.295).

The study in the CPB/DHCA subgroup showed no significant difference in AKI risk between a 3-day time interval and an interval of more than 3 days, with an adjusted OR of 0.35 (95% CI, 0.17-0.73; *P* = 0.005).

## Discussion

This meta-analysis is the first to evaluate the impact of the time interval between CAG and cardiac surgery on postoperative AKI incidence. Our results suggest that a ≤1-day time interval significantly increases postoperative AKI. This review provides important evidence that may resolve the ongoing controversy arising from previous studies. For instance, Ko et al published a series of 2133 consecutive patients who underwent cardiac surgery but found no association between the time interval from angiography to surgery and the incidence of postoperative AKI [9]. Conversely, Ranucci et al recently reported that surgery on the same day as angiography significantly increases the risk of AKI, after risk-adjustment in a total of 4440 consecutive patients [14]; these results closely resemble those of the present meta-analysis. The validity of these results supports the idea that the incidence of postoperative AKI can be contained by limiting the practice of performing cardiac surgery on the same day as angiography [14].

The 5 studies of a 3-day time interval that were included in this meta-analysis exhibited severe heterogeneity. After considering the obvious difference of the use of DHCA, a risk factor of AKI [23,24], we divided the studies into to two subgroups: the CPB subgroup, included 4 studies; and the CPB/DHCA subgroup, only 1. Once we removed the study that used DHCA, the heterogeneity of the CPB subgroup disappeared.

Analysis of the CPB subgroup determined that a time interval of 3 days or less between CAG and cardiac surgery significantly increases postoperative AKI. This time interval includes surgeries performed 1, 2, and 3 days after CAG. Without consideration of the effect of a time interval of 1 day or less, it is difficult to evaluate the effect of 2-day and 3-day time intervals on the incidence of postoperative AKI. In fact, the pooled effect is largely based on data on the incidence of AKI from 2 studies, which contributed to more than 90% of the total weight. In the cohort study by Ko et al, the number of days between CAG and cardiac surgery was not a predictor of postoperative AKI after the data were adjusted for confounding factors (OR, 0.99; 95% CI, 0.99 - 1.00; *P* = 0.41) [9]. In the study by Mehta et al, cardiac surgeries performed 2 days and 3 days after CAG were not associated with an increased risk of AKI when compared with those performed later (OR, 1.26; 95% CI, 0.94 – 1.74; and OR, 1.11; 95% CI, 0.77 – 1.59) [22]. Accordingly, the significant association between a time interval of 3 days or less and increased risk of AKI may have resulted from inclusion of data from a time interval of 1 day or less, not from inclusion of data from 2-day or 3-day time intervals.

The lack of association between CAG on preoperative days 1 through 3 and increased risk of AKI in the one study of the CPB/DHCA subgroup seems controversial, given that the pooled effect of the CPB subgroup did show a significant association (adjusted OR, 0.35; 95% CI, 0.17-0.73; *P* = 0.005) [10]. However, the CPB/DHCA subgroup had a much more higher incidence of AKI, about 31% if defined by the RIFLE criteria, while, the highest incidence of the other studies was only 18% if defined by the RIFLE criteria, or 32% if defined by the AKI network criteria [9]. W e interpret this to mean that DHCA plays an important role in postoperative AKI. Moreover, the increased risk of AKI due to DHCA would probably “dilute” the difference between any CPB/DHCA subgroups containing studies that report data from different time intervals.

Our review has several limitations that must be considered for accurate interpretation of the reported effects. First, this observational meta-analysis was based on a limited number of cohort and case-control studies and was short of randomized trials and a large scale of comprehensive clinical trials. Accordingly, the potential confounding factors such as age, mellitus diabetes, type of disease, type or complexity of the operation, bypass time and the use of DHCA were unequally distributed. The impact of this bias on the estimated effects presented in this review is unknown, even after adjustment. To address this issue, the methods we used to select studies and analyze pooled data were in accordance with the MOOSE guideline [25] and current recommendations for meta-analysis of observational trials. Additionally, we used a funnel plot analysis and Eggers' test to exclude publication bias. Secondly, this review was limited by the use of different definitions of AKI, although Haase et al reported that the incidence of postoperative AKI in patients with cardiac surgery was similar when AKI was defined according to either the RIFLE or the AKI Network classification [26]. We attempted to mitigate this bias to some extent by adopting OR as the summary statistic. Lastly, our review did not account for differences in study quality, since the rating of methodological quality was “good” for all included studies.

## Conclusions

The results of this meta-analysis strongly support an association between a ≤ 1-day time interval from CAG to cardiac surgery and increased risk of AKI. The similar association between a day time interval of 3 days or less and risk of AKI probably resulted from inclusion of data from an interval of a day or less. We propose that the delay of cardiac surgery until 24 hours after CAG can potentially decrease postoperative AKI. In the future it will be necessary to evaluate the risk of a short time interval between CAG and cardiac surgery in a randomized trial, and clarify the other AKI risk factors in the setting of a short interval between CAG and cardiac surgery. This would help to adjust the estimation of appropriate individual risk and to optimize the flow of treatment in patients who require diagnostic preoperative coronary angiography.

## Abbreviations

CAG: Coronary angiography; AKI: Acute kidney injury; ORs: Odds ratios; CI: Confidence interval; CABG: Coronary artery bypass grafting; CPB: Cardiopulmonary bypass; DHCA: Deep hypothermic circulatory arrest.

## Competing interests

The authors declare that they have no competing interests.

## Authors’ contributions

ZQ and HY designed the study. HY and LZ carried out studies searching and performed the eligibility assessments. HY and SY evaluated the qualities of the included studies and carried out data extracting. ZQ, CJ, LZ and SC analyzed and interpreted the data. HY drafted the manuscript. ZQ, LZ, CJ and SC made critical revision of the manuscript for important intellectual content. All authors read and approved the final manuscript.
